# Prognostic analysis of m6A-related genes as potential biomarkers in idiopathic pulmonary fibrosis

**DOI:** 10.3389/fgene.2022.1059325

**Published:** 2022-11-29

**Authors:** Zhiqiang Wang, Lanyu Shen, Junjie Wang, Jiaqian Huang, Huimin Tao, Xiumin Zhou

**Affiliations:** ^1^ Department of Oncology, The First Affiliated Hospital of Soochow University, Suzhou, China; ^2^ Department of Biochemistry and Molecular Biology, Medical College, Soochow University, Suzhou, China

**Keywords:** N6-methyladenosine (m6A), WGCNA, m6A-related genes, prognosis risk model, IPF

## Abstract

Idiopathic pulmonary fibrosis (IPF) is a progressive, fatal lung disease with limited treatment options. N6-methyladenosine (m6A) is a reversible RNA modification and has been implicated in various biological processes. However, there are few studies on m6A in IPF. This project mainly explores the prognostic value of m6A-related genes as potential biomarkers in IPF, in order to establish a set of accurate prognostic prediction model. In this study, we used GSE28042 dataset in GEO database to screen out 218 m6A-related candidate genes with high IPF correlation and high differential expression through differentially expressed gene analysis, WGCNA and m6A correlation analysis. The genes associated with the prognosis of IPF were screened out by univariate Cox regression analysis, LASSO analysis, and multivariate Cox regression analysis, and the multivariate Cox model of prognostic risk of related genes was constructed. We found that RBM11, RBM47, RIC3, TRAF5 and ZNF14 were key genes in our model. Finally, the prognostic prediction ability and independent prognostic characteristics of the risk model were evaluated by survival analysis and independent prognostic analysis, and verified by the GSE93606 dataset, which proved that the prognostic risk model we constructed has a strong and stable prediction efficiency.

## 1 Introduction

Pulmonary fibrosis (PF) is a chronic, progressive tissue repair response, which leading to irreversible scarring and lung remodeling (King et al., 2011). PF can occur secondary to certain predisposing factors or diseases, such as radiation (He et al., 2019), asbestos (Pira et al., 2018), silica (Cao et al., 2020), drugs (Della Latta et al., 2015), autoimmune diseases (Fischer and Distler, 2019), etc. However, some patients with PF without a clear cause, which is called idiopathic pulmonary fibrosis (IPF). IPF is a chronic, age-related interstitial lung disease (ILD) characterized by excessively deposition of extracellular matrix (ECM) protein and irreversible loss of lung function, causing progressive respiratory failure (Richeldi et al., 2017; Barratt et al., 2018). The pathogeny of IPF is still unknown, but it likely related to heredity and environment. There are large regional differences in the incidence of IPF, ranging from 0.35 to 1.30 per 100,000 individuals in Asia–Pacific countries, 0.09 to 0.49 per 100,000 individuals in Europe, and 0.75 to 0.93 per 100,000 individuals in North America (Maher et al., 2021). IPF tends to occur in men between 40 and 50 years of age and has a poor prognosis. The average life expectancy of untreated IPF patients is only 3–5 years, and most patients die of acute exacerbations of IPF or respiratory failure. Actually, acute exacerbations of IPF can occur at any time during the course of the disease and are associated with extremely high mortality (Spagnolo and Wuyts, 2017). Although two antifibrotic drugs, nintedanib and pirfenidone, have been shown to delay the progression of IPF, there is currently no drug that can cure IPF (Raghu et al., 2015).

Epigenetics usually refers to the heritable modification of genetic material without changing gene sequence, including DNA methylation, RNA methylation, histone modification, chromosome remodeling, etc., which plays an important role in various diseases and tumors (Berger et al., 2009). At present, more than 100 kinds of RNA (mRNA, lncRNA, snRNA, etc.) have been found post-transcriptional modifications, among which N6-methyladenosine (m6A) is the most common (Yue et al., 2015; Boccaletto et al., 2018). M6A RNA modification is a dynamic and reversible post-transcriptional modification process mediated by m6A WER proteins (methyltransferase “writers”, demethylase “erasers”, binding proteins “readers”), which plays a crucial regulatory role in RNA metabolism, splicing, translation and other processes (Wang et al., 2020). Previous studies have shown that m6A is widely involved in the development of various diseases, such as pneumonia, lung cancer, colorectal cancer, breast cancer, nasopharyngeal cancer, systemic lupus erythematosus, etc. (Li et al., 2018; Chang et al., 2020; Yue et al., 2020; Maher et al., 2021; Meng et al., 2021; Li et al., 2022). For example, Li et al. (2021) found that SNHG4 promoted LPS-induced inflammation by inhibiting METTL3-mediated m6A level of STAT2 mRNA. And research pointed out that overexpressed FTO enhanced the expression of MZF1 by reducing the m6A modification level and stability of MZF1 mRNA, thereby promoting the development of lung cancer (Liu et al., 2018). Similarly, enhanced activity of methyltransferase METTL3 increased the m6A modification level of JUNB mRNA and accelerated the progression of TGF-β-induced lung adenocarcinoma (LUAD) (Wanna-Udom et al., 2020). These studies indicated that RNA methylation regulators could affect the development of the above diseases by regulating the m6A modification of RNA. M6A-related genes can also be used as diagnostic and prognostic markers for lung diseases. For example, studies found that m6A-related genes (EGFR, RFXAP, KHDRBS2, ADAMTS6, etc.) were determined to be associated with overall survival (OS) in patients with LUAD, in which RFXAP and KHDRBS2 exhibited independent prognostic value (Sun et al., 2021). Additionally, Jia et al. (2022) showed that three m6A-related genes (FAM71F1, MT1E, and MYEOV) were identified as prognostic genes in Lung Squamous Carcinoma (LUSC). However, there are few reports on m6A methylation modification in the occurrence and development of IPF. Therefore, it is of great significance to explore m6A-related genes and construct IPF-related prognostic risk model to assist in judging the progression and prognosis of IPF.

Weighted gene co-expression network analysis (WGCNA) is a comprehensive analysis technique based on biological network, which can identify a class of genes (or proteins) that are co-expressed, and cluster genes with similar expression patterns through algorithms into different modules, analyze the association between modules and characteristic traits or phenotypes, use clustering modules to associate with phenotypes to build a co-expression network, and explore the core genes (or proteins) in the modules, so as to provide ideas for exploring the molecular mechanism of diseases (Presson et al., 2008; Yin et al., 2018). Compared with microarray and high-throughput sequencing analysis, WGCNA is suitable for multiple statistical tests to analyze the correlation between genes and avoid losing the trend information of genes according to a fixed threshold screening.

The Cox proportional hazards model is essentially a regression model commonly used in medical research statistics to study the association between a patient’s survival time and one or more predictor variables (Cox, 1972). It is applicable to quantitative predictor variables and categorical variables. It mainly includes univariate and multivariate Cox regression analysis. Univariate Cox analysis is usually used to remove collinearity, but may lead to synergistic effects caused by other variables, so multivariate Cox regression is performed to correct other factors, which is often used for data modeling in survival analysis (Huang and Liu, 2006; Li et al., 2016).

**FIGURE 1:**
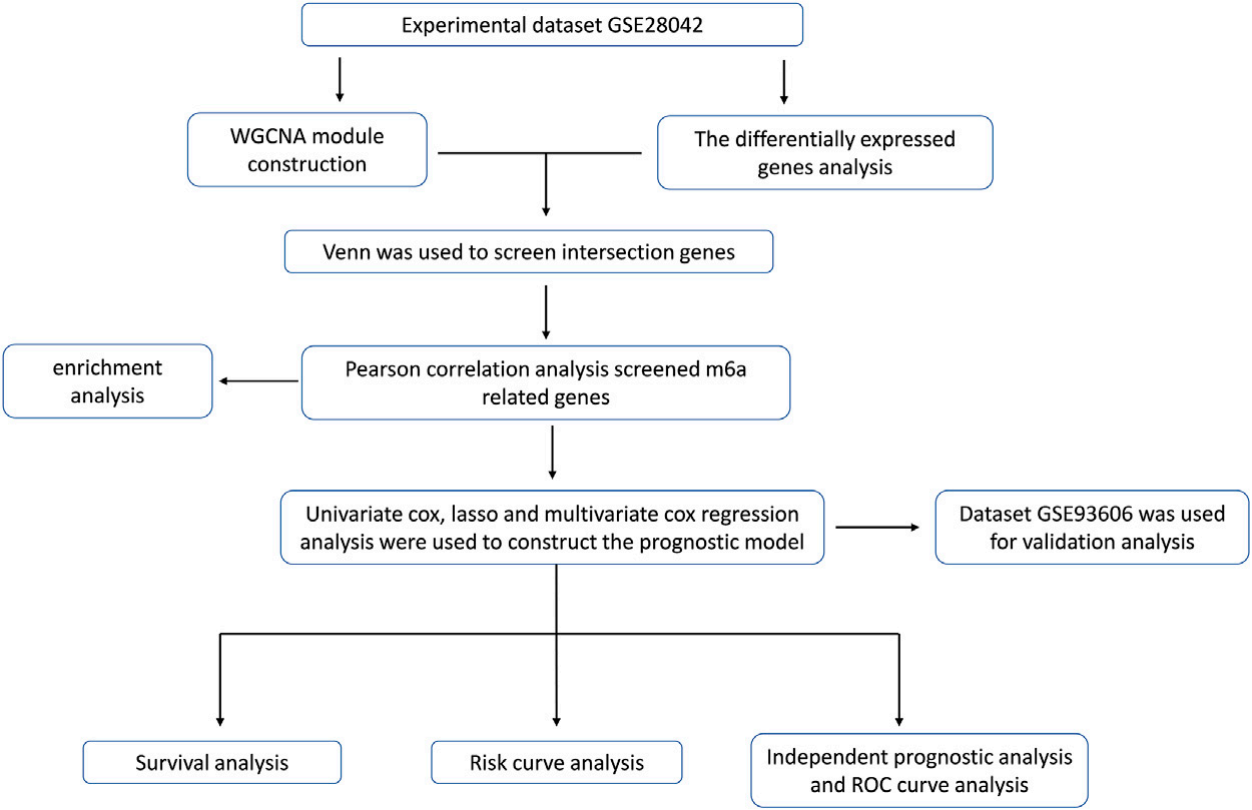
The workflow for prognostic analysis of m6A-related genes as potential biomarkers for idiopathic pulmonary fibrosis.

In this paper, the microarray data GSE28042 was downloaded from the Gene Expression Omnibus (GEO) database, and the gene expression profiles of peripheral blood mononuclear cell (PBMC) and the corresponding clinical data of 75 IPF samples and 19 normal samples were obtained. Through the analysis of differentially expressed genes, WGCNA and m6A correlation analysis method, a group of m6A-related candidate genes with high IPF correlation and differential expression were screened. The genes associated with the prognosis of IPF were screened out by univariate Cox regression analysis, LASSO analysis, and multivariate Cox regression analysis, and the multivariate Cox model of prognostic risk of related genes was constructed. Finally, the prognostic predictive ability and independent prognostic characteristics of the risk model were evaluated by survival analysis and independent prognostic analysis, and verified by GSE93606 dataset, which is intended to provide a basis for prognostic prediction of IPF patients (Figure 1).

## 2 Materials and methods

### 2.1 Data collection and processing

First, we searched the GEO database (https://www.ncbi.nlm.nih.gov/geo/) for keywords such as “idiopathic pulmonary fibrosis”, “survival”, “blood”, etc. Then, by combining samples for survival information, we eventually included the GSE28042 and GSE93606 datasets into the study. GSE28042 was used as the experimental dataset and GSE93606 was used as the validation dataset. The GSE28042 dataset contains the gene expression profiles of peripheral blood mononuclear cell (PBMC) and their corresponding clinical data of 75 IPF patients and 19 healthy people. The probes were converted to corresponding gene symbols by referring to the annotation information of the GPL6480 [Agilent-014850 Whole Human Genome Microarray 4 × 44K G4112F (Probe Name version)] platform. The GSE93606 dataset contains peripheral whole blood gene expression profiles and corresponding clinical data of 60 IPF patients and 20 healthy subjects. The probes were converted to the corresponding gene symbols by referring to the annotation information of GPL11532 [Hugene-11-ST] Affymetrix Human Gene 1.1 ST Array [transcript (Gene) version] platform.

### 2.2 Construction of weighted gene co-expression network analysis

In order to explore the modules and genes related to the clinical characteristics of healthy people and IPF patients, the data of GSE28042 were analyzed by using the WGCNA package of R language, and the samples were clustered. In order to ensure the reliability of the results, we analyzed the samples and removed the samples that were not clustered, that is, the outlier samples. In order to ensure that the network conforms to the scale-free network distribution, the “pickSoftTreshold” function in the WGCNA package is used to calculate the correlation coefficient of β value and the mean of gene connectivity, and the appropriate soft threshold β is selected to make the network conform to the standard of scale-free network. Then, the modules were clustered with a minimum cluster of 100 genes and a cut height of 0.25. Finally, the gene significance (GS) and module membership (MM) were calculated and correlated with clinical traits. The two modules with the highest correlation with IPF were selected, and the genes in the modules were further analyzed. Genes in the co-expression module have high connectivity and genes in the same module may have similar biological functions.

### 2.3 DEG analysis

Using R language (R) 4.0.3 limma package to analyze the gene differences between the gene expression matrix of peripheral blood monocytes of healthy people and IPF patients. Set the screening criteria as |log_2_FC| >0.5, *p* < 0.05 (correction method is FDR). The up-and down-regulated genes were represented by mapping volcanoes.

### 2.4 Screening of differentially expressed genes associated highly with idiopathic pulmonary fibrosis

The common genes obtained by WGCNA analysis and DEG analysis were defined as IPF highly correlated differential genes. Use the Venn diagram (https://bioinfogp.cnb.csic.es/tools/venny/index.html) to show all the differentially expressed genes associated highly with IPF.

### 2.5 Identification of m6A-related candidate genes

The cor () and cor. test () functions of R language were used to calculate the correlation between the expression levels of 23 m6A regulators (METTL3, METTL14, METTL16, WTAPI, VIRMA, ZC3H13, RBM15, RBM15B, YTHDC1, YTHDC2, YTHDF1, YTHDF2, YTHDF3, HNRNPC, FMR1, LRPPRC, HNRNPA2B1, IGFBP1, IGFBP2, IGFBP3, RBMX, FTO, ALKBH5) and the expression levels of IPF highly correlated differential genes and calculate the *p* value (Deng et al., 2018; Chen et al., 2019). The genes significantly associated with either m6A regulator (| Pearson R | > 0.5 and *p* < 0.05) was defined as candidate genes related to m6A.

### 2.6 Gene function and pathway enrichment analysis

The online website Metascap (https://metascape.org/gp/index.html) was used to analyze the module function and pathway enrichment of m6A-related candidate genes to further explore the biological functions of these genes. GO analysis was used to annotate the functions of genes and their products in three aspects, including biological process (BP), molecular function (MF) and cellular component (CC). KEGG database is a collection of information about genes, proteins, chemical components and their interactions, reactions and relationship networks to annotate gene functions and metabolic pathways.

### 2.7 Construction of prognostic risk model and independent prognostic analysis

A series of m6A-related prognostic genes were screened by univariate Cox regression analysis (KM < 0.05, *p* < 0.05), and further screened by LASSO regression method. Then, the prognosis model was constructed by multivariate Cox regression analysis, and the forest map was drawn. The Kaplan-Meier method of the “survival” function package was used to analyze the survival of the screened genes, and the survival curve was drawn.

The median prognostic risk value was set as a threshold. According to this threshold, samples from patients with IPF patients were divided into low-risk and high-risk groups. The distribution of risk grades between the low-risk group and the high-risk group was plotted as a scatter plot. The survival status and survival time of patients in the two different risk groups were also plotted as a scatter plot. Then the Kaplan-Meier method was used to draw survival curves for the risk models.

Clinical traits and risk values were compared with survival time and survival status. Independent prognostic analysis was conducted to test the prognostic ability of the prognostic risk model, and to observe whether the prognostic model can be independent of other clinical traits and whether it has independent prognostic characteristics of IPF. The R package “timeROC” was used to draw time-dependent ROC curves and “survivalROC” was used to verify the accuracy of the prognostic risk model. The ROC curve was drawn to predict the accuracy of the model, and the accuracy was judged by the area under the curve.

### 2.8 Statistical analysis

In this study, the R (version 4.2.0) and RStudio software were utilized to carry out the statistical analysis and figure preparation. *p*-values less than 0.05 were defined as statistically significant.

## 3 Results

### 3.1 WGCNA module construction and selection of modules with high correlation with idiopathic pulmonary fibrosis

**FIGURE 2:**
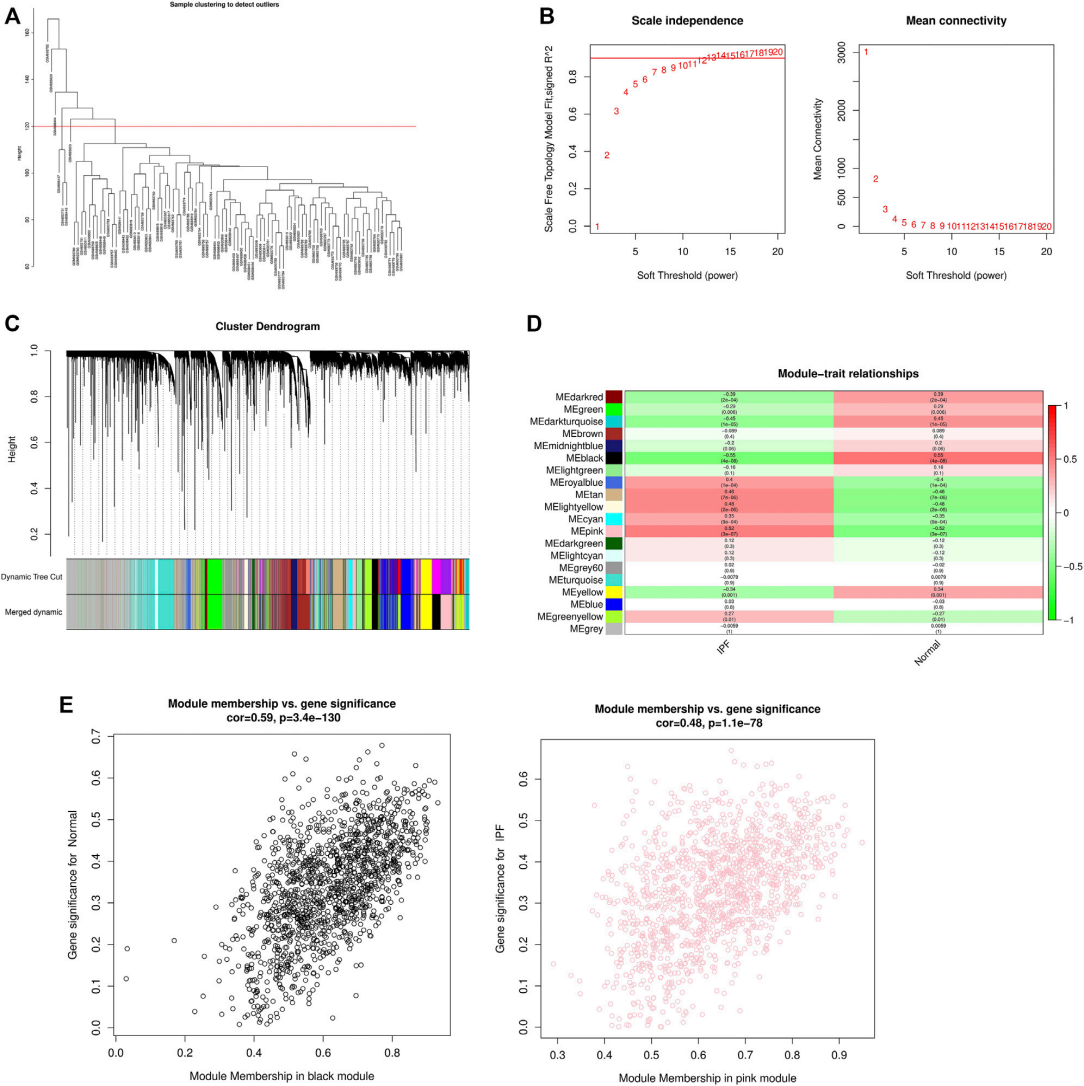
WGCNA module construction and selection of modules with high correlation with IPF. **(A)** Sample clustering diagram (delete 7 outlier samples by setting the height to 120); **(B)** Determination of the optimal soft threshold (in the process of module selection, the adjacency matrix is converted into a topology matrix, and the optimal soft threshold β = 10 is determined); **(C)** Cluster tree of co-expressed gene modules (similar genes are grouped into the same module through dynamic splicing and cluster analysis); **(D)** The correlation between gene modules and clinical information (The redder the color, the higher the positive correlation; the greener the color, the higher the negative correlation. Numbers in the figure are Pearson’s correlation coefficient, and corresponding *p*-values are in parentheses); **(E)** The correlation between Black and Pink modules and IPF is represented by scatter plot.

WGCNA analysis was performed using gene expression matrix. After setting the high degree to 120, 7 outlier samples (GSM693752, GSM693820, GSM698444, GSM698447, GSM698445, GSM693751, GSM693823) were removed. Finally, 71 IPF samples and 16 normal samples were analyzed later (Figure 2A). When the scale-free topological fitting index R2 reaches 0.9, the appropriate β value is chosen as 10 (Figure 2B). The dynamic clipping tree algorithm was provided to segment the modules and construct the network diagram. Cluster analysis was performed on the modules and the modules with similarity greater than 0.75 were merged into new modules, in which the minimum module had 100 genes and the clipping height was 0.25 (Figure 2C). On this basis, the WGCNA method based on sequence free network was used to modularize genes, and the topological overlap matrix between all genes was described by heat map, and the relationship between sample features and modules was analyzed. The colors corresponding to the modules are darkred, green, darkturquoise, brown, midnightblue, black, lightgreen, royalblue, tan, lightyellow, cyan, pink, darkgreen, lightcyan, grey60, turquoise, yellow, blue, greenyellow, grey. Among them, the grey module is the gene that cannot be clustered to other modules, so it will not be analyzed in the subsequent analysis (Figure 2D). Key modules were identified according to the correlation coefficient between module features and traits, in which the black module had the highest positive correlation (cor = 0.59, *p* < 3.4e-130), and the pink module had the highest negative correlation (cor = 0.48, *p* < 1.1e-78), and finally determined that the black module and the pink module were the two modules with the highest degree of IPF correlation. A scatter plot was used to represent the correlation between black or pink modules and IPF, and a total of 2729 genes were found (Figure 2E).

### 3.2 The differentially expressed genes between idiopathic pulmonary fibrosis samples and normal samples were screened

**FIGURE 3:**
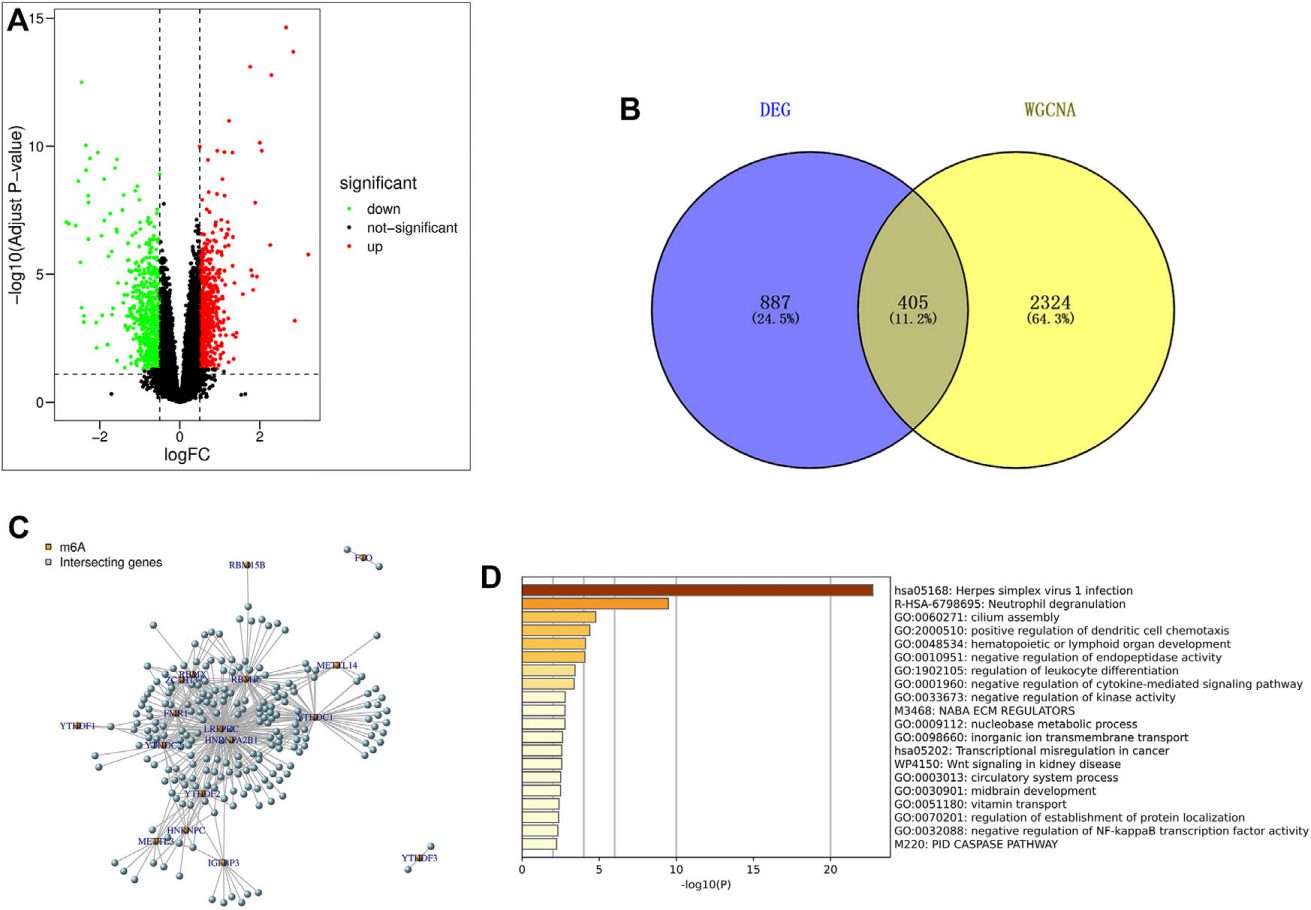
Screening and enrichment analysis of m6A related candidate genes. **(A)** Volcano map of differentially expressed genes (red are up-regulated genes, green are down-regulated genes, black are non-differentially expressed genes); **(B)** The genes screened by DEG and WGCNA were intersected by Venn diagram, and IPF highly correlated differentially expressed genes were obtained; **(C)** Pearson correlation analysis was used to screen out m6A-related candidate genes in IPF; **(D)** GO and KEGG enrichment analysis were performed for m6A related candidate genes.

Using the limma package in R language to screen differentially expressed genes, based on |log_2_FC|>0.5 and *p* < 0.05 (correction method is FDR) as the threshold, the differential genes in the IPF patients and healthy population samples in the GSE28042 dataset were screened. A total of 1292 differentially expressed genes were found, of which 606 genes were up-regulated and 686 were down-regulated. The results of differentially expressed genes were used to construct a volcano plot, where red represents up-regulated genes, green represents down-regulated genes, and black represents genes defined as non-differential (Figure 3A).

### 3.3 Screening of IPF highly correlated differentially expressed genes

The 2729 genes in Black and Pink modules obtained by WGCNA analysis were highly correlated with IPF, and the 1292 genes obtained by DEG analysis were significantly different. Therefore, a total of 405 genes were obtained by taking the intersection of the two genes through Venn diagram, and these genes were defined as IPF highly correlated differentially expressed genes (Figure 3B).

### 3.4 Screening and enrichment analysis of m6A-related candidate genes

Pearson correlation analysis was used to screen out 218 candidate genes related to m6A from IPF highly correlated differentially expressed genes (|Pearson R|>0.5, *p* < 0.05) (Figure 3C). At the same time, the online website Metascap (https://metascape.org/gp/index.html) was used to analyze the candidate genes related to m6A. The results showed that the candidate genes mainly focused on the pathways of herpes simplex virus type I infection, neutrophil degranulation, cilia assembly and so on (Figure 3D).

### 3.5 Construction of prognostic risk model

**FIGURE 4:**
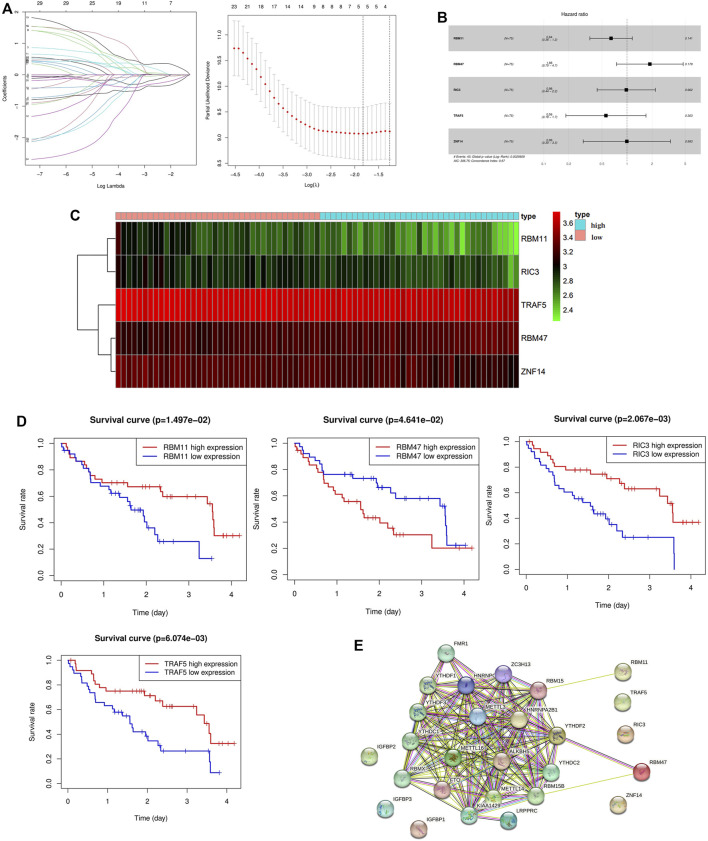
Screening of key genes associated with IPF prognosis. **(A)** LASSO regression analysis screened 5 genes associated with prognosis; **(B)** Multivariate Cox regression analysis of the effect of five key genes RBM11, RBM47, RIC3, TRAF5, ZNF14 on the prognosis of patients with IPF; **(C)** Expression levels of key candidate genes in different IPF samples; **(D)** Kaplan-Meier survival analysis of key genes; **(E)** Protein interactions between five key genes and 23 m6A regulators.

30 genes associated with IPF prognosis were screened out from 218 m6A-related candidate genes by univariate Cox method (Table 1), and 5 genes associated with IPF prognosis were further screened by LASSO method (Figure 4A). On this basis, further multivariate Cox regression analysis showed that RBM11, RBM47, RIC3, TRAF5 and ZNF14 candidate genes had significant impact on the prognosis of IPF patients (Figure 4B). These five genes were used to construct a multivariate Cox model of prognostic risk in IPF patients, riskscore= (−0.44084*RBM11)+ (0.631579*RBM47) + (−0.01935*RIC3) + (−0.58291*TRAF5) + (−0.00528*ZNF14) (Table 2). The expression heat map and survival analysis of these five genes were displayed (Figures 4C,D). Among them, the survival rate was low when RBM47 was highly expressed, while the survival rate was high when RBM11, RIC3, TRAF5, and ZNF14 were highly expressed. The protein-protein interactions between 5 genes and 23 m6A regulators were analyzed by the STRING database (https://cn.string-db.org/), and it was found that there were obvious protein-protein interactions between RBM11, RBM47 and m6A regulators (Figure 4E). In addition, m6A-Atlas (http://rnamd.org/m6a/) also showed that the five key genes had m6A sites, which increased the credibility of the research content.

**TABLE 1:** The univariate Cox regression analysis demonstrating 30 genes associated with IPF prognosis.

ID	HR	*p* value
ACPP	2.819365	0.007666706
ADAP2	3.162390	0.010084724
BEST1	2.767257	0.004894885
BIRC3	0.380656	0.001404292
C19orf59	2.313709	0.003886102
CLEC2D	0.330340	0.002016299
CLK1	0.274811	0.005419468
CLK4	0.201311	0.00567289
DOCK5	3.361948	0.00399794
EFHA2	0.556492	0.007436865
FAM161A	0.571926	0.045819138
FRAT1	2.398299	0.009385627
JDP2	2.102222	0.005909982
KIAA1147	0.418338	0.02874775
KLF12	0.438746	0.007658451
LRBA	0.432600	0.048624944
MIDN	2.336528	0.03280022
RBM11	0.465034	0.0003472
RBM47	3.284265	0.001557636
RIC3	0.410807	0.00061674
SACS	0.520899	0.04322521
SLC38A1	0.327794	0.002712488
SLC8A1	2.434524	0.012780222
TIMP2	2.492529	0.030980637
TRAF5	0.257449	0.000397483
TTC18	0.314697	0.001033081
ZNF14	0.295492	0.000512298
ZNF30	0.380889	0.007052629
ZNF529	0.298259	0.000842414
ZNF573	0.258771	0.001805436

**TABLE 2:** The result of multivariate COX regression analysis.

ID	COEF	HR	HR.95L	HR.95H	*p* value
RBM11	−0.44084	0.643493	0.357941	1.156848	0.140723
RBM47	0.631579	1.880578	0.748600	4.724255	0.178993
RIC3	−0.01935	0.980836	0.438145	2.195713	0.962464
TRAF5	−0.58291	0.558274	0.184302	1.691076	0.302605
ZNF14	−0.00528	0.994734	0.297910	3.321466	0.993152

### 3.6 Survival analysis and independent prognostic analysis

**FIGURE 5:**
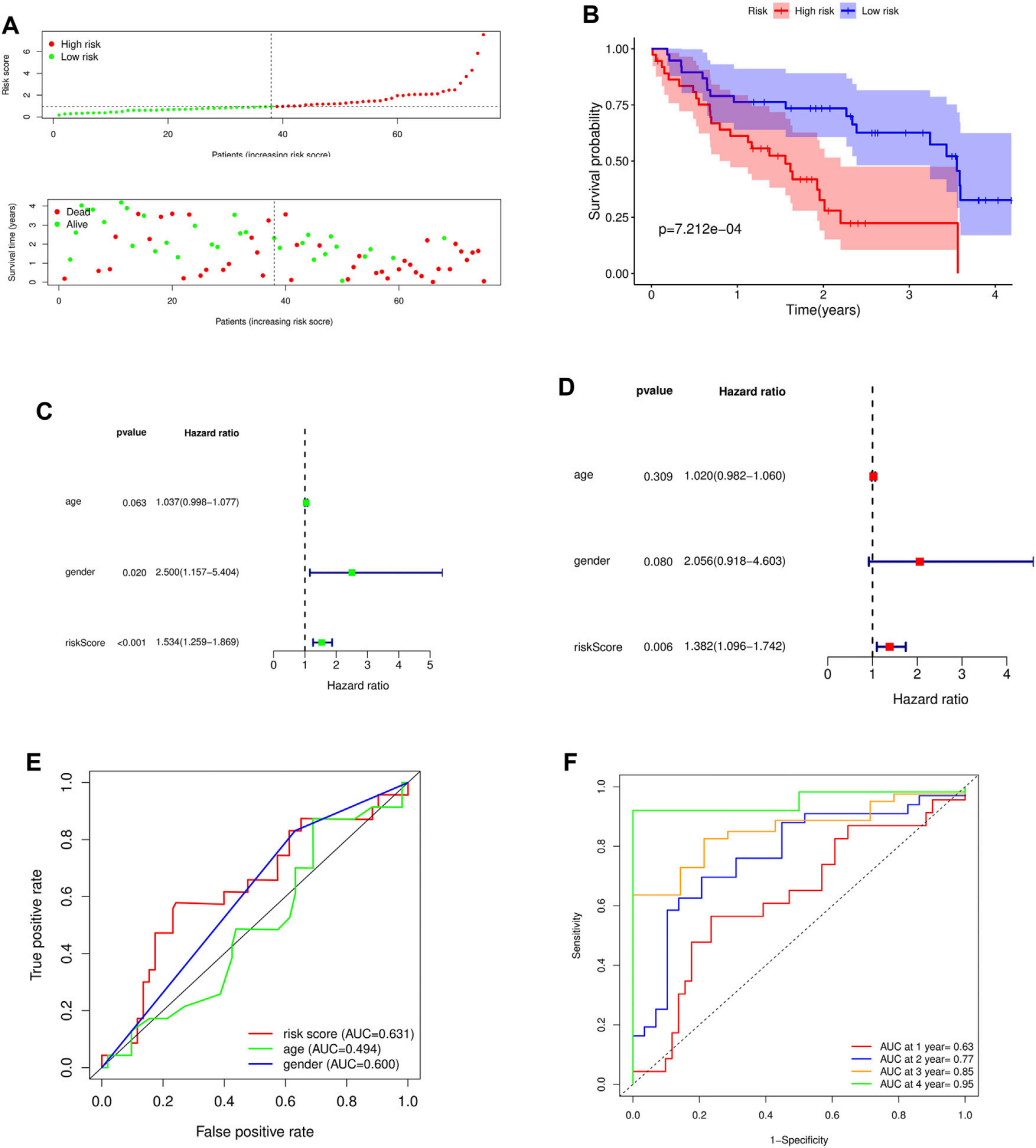
Survival analysis and independent prognostic analysis of the prognostic risk model. **(A)** Distribution of patients in different risk groups and risk levels; **(B)** Overall survival curve of the model; **(C)** Univariate independent prognostic analysis; **(D)** Multivariate independent prognostic analysis; **(E)** ROC curve of different factors (riskscore, age, gender); **(F)** ROC curve of different years (1, 2, 3 and 4 years).

To further verify the predictive ability of the model for prognosis, we took the median risk value of patients as the threshold, divided patients into high risk group and low risk group, and analyzed the survival status and survival time of patients in two different risk groups (Figure 5A). And through the survival curve, it was found that the survival rate of high-risk patients was low, while the survival rate of low-risk patients was high, which preliminarily demonstrated the correctness of the model (Figure 5B).

To further assess whether the risk model for these 5 key genes has independent prognostic features of IPF, we performed an independent prognostic analysis. We performed univariate and multivariate independent prognostic analyses for the above five key genes, respectively, indicating that the risk model of the five key genes was independent of other clinicopathological parameters (gender, age) (Figures 5C,D).

By analyzing the prognostic risk model and drawing the ROC curve, it was found that compared with other factors, the AUC value of riskscore was greater than that of other factors (age and gender) (Figure 5E). By plotting the time-dependent ROC curve of the prognostic risk model, it can be found that although the AUC value in the first year was low (AUC at 1 year = 0.63), the AUC value gradually increased with time (AUC at 2 years = 0.77, AUC at 3 years = 0.85, AUC at 4 years = 0.95) (Figure 5F). This indicates that the accuracy of our prognostic model is good.

### 3.7 Validation of prognostic risk model

**FIGURE 6:**
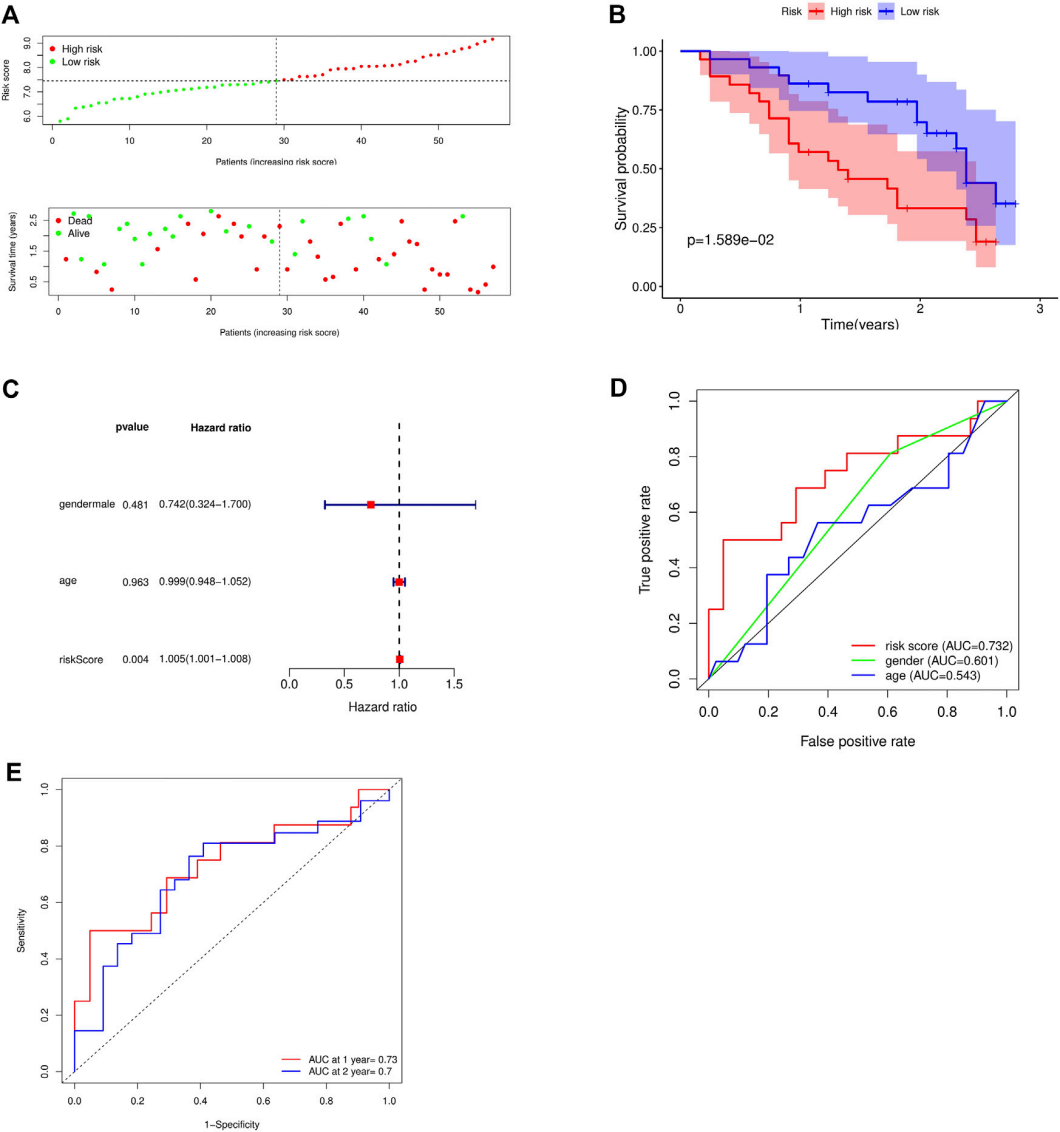
Validation of the accuracy of the prognostic model using the GSE93606 dataset. **(A)** Distribution of patients and risk levels in different risk groups; **(B)** Overall survival curve of the model; **(C)** Multivariate independent prognostic analysis; **(D)** ROC curve of different factors (age and riskscore); **(E)** ROC curve of different years (1, 2 years).

The GSE93606 dataset was used as the validation dataset to validate our prognostic risk model by survival analysis and independent prognostic analysis. In the validation dataset, survival analysis verified that high-risk patients had a low survival rate, while low-risk patients had a high survival rate (Figures 6A,B). Multivariate prognostic analysis verified that the prognostic risk model was independent of other clinicopathological parameters (gender and age) (Figure 6C). ROC curve verified the accuracy of the prognostic risk model (Figures 6D,E). These results indicate that the prognostic risk model has strong and stable predictive efficiency.

## 4 Discussion

The etiology of IPF is still not fully understood, but some studies have shown that its pathogenesis may be related to the abnormal damage and repair of alveolar epithelial cells, epithelial-to-mesenchymal transition (EMT), fibroblast-to-myofibroblast transformation (FMT), and inflammatory response (King et al., 2011). Worldwide, the incidence and mortality of IPF are on the rise. Lung transplantation is the only treatment for IPF that can prolong life expectancy (Kumar et al., 2018). Unfortunately, IPF patients without lung transplantation have a short median survival time. M6A is the most abundant post-transcriptional modification in mRNA and long non-coding RNA (lncRNA) in most eukaryotes. In addition, studies have reported that m6A is involved in post-transcriptional modification, cell differentiation, cell recoding, cell stress and other processes, and plays an important role in lung diseases such as lung cancer, pulmonary hypertension and chronic obstructive pulmonary disease through various mechanisms. However, there are few studies on m6A in IPF. Therefore, it is necessary to explore the prognostic value of m6A-related genes in IPF and establish a set of prediction models for evaluating the survival time of IPF and improving the prognosis of patients.

In this study, we downloaded GSE28042 dataset from GEO database, which included peripheral blood monocyte cell gene expression profiles and their corresponding clinical information of 75 IPF samples and 19 normal samples, and analyzed the obtained data. The gene expression matrix was used for differential gene analysis, and 606 up-regulated genes and 686 down-regulated genes were screened. The correlation between each module and the trait was obtained by WGCNA analysis combined with correlation heat map. The black and pink modules with the highest positive and negative correlations were selected, and 405 intersection genes were obtained by intersection of the DEG and the module genes with the highest correlation in the selected WGCNA. Then, 218 m6A-related candidate genes were screened out from the 405 IPF highly correlated differentially expressed genes by Pearson correlation analysis, and the enrichment analysis of these genes showed that the above genes were mainly enriched in herpes simplex virus type Ⅰ(HSV-1) infection, neutrophil degranulation, ciliary assembly and other pathways. Studies have shown that chronic viral infections, mainly herpes virus infections, may contribute to the development of IPF. And HSV-1 is a kind of herpes virus, it can enter the alveoli through the respiratory tract and spread with the blood, resulting in focal necrotizing pneumonia, followed by diffuse pulmonary fibrosis (Luyt, 2020). Neutrophil degranulation is one of the important links that neutrophils participate in the inflammatory response. As inflammatory cells, neutrophils participate in the progression of PF by promoting the proliferation of fibroblasts and enhancing the differentiation of myofibroblasts (Gregory et al., 2015; Klopf et al., 2021). Cilia is an organelle protruding from the cell surface. The abnormal structure and function of cilia can cause various diseases, such as bronchiectasis and reproductive infertility (Jain et al., 2012; Girardet et al., 2019). Moreover, studies have shown that pulmonary fibrosis is associated with bronchiectasis (Fitzgerald et al., 2017). The above relevant findings suggest that the m6A-related candidate genes screened were closely related to the occurrence and development of PF. Therefore, we hypothesized that the m6A-related candidate genes were associated with IPF.

In order to explore the role of m6A-related candidate genes in the prognosis of IPF, we screened out 30 genes associated with patient prognosis by univariate Cox analysis, and then screened out 5 key genes (RBM11, RBM47, RIC3, TRAF5, ZNF14) by LASSO analysis and multivariate Cox analysis. The above studies indicate that the five key genes and 23 m6A regulators are significantly correlated and modified by their regulation. This regulation can be direct or indirect, but its specific mechanism is still unknown. The results of protein-protein interaction analysis also showed that RBM11 and RBM47 had protein-protein interactions with m6A regulators, and the m6A-Atlas analysis showed that all five key genes had m6A sites (Tang et al., 2021), which added confidence to our results. We construct a riskscore model as an indicator to predict the prognosis of IPF [riskscore = (−0.44084*RBM11) + (0.631579*RBM47) + (−0.01935*RIC3) + (−0.58291*TRAF5) + (−0.00528*ZNF14)], and then survival analysis was performed to assess the effect of the above genes on the prognosis of IPF patients. The results of single-gene survival analysis showed that high expression of RBM11, RIC3, TRAF5, ZNF14 was associated with good prognosis of IPF, while high expression of RBM47 was associated with poor prognosis; overall survival analysis of the risk prognostic model showed that high-risk patients had poor survival, while low-risk patients had higher survival, which preliminarily indicated the correctness of the model. Simultaneous univariate and multivariate independent prognostic analyses indicated that the risk model for these five key genes was independent of other clinicopathological parameters (gender, age). TRAF5 is an important signal transducer for a wide range of TNF receptor superfamily members, and it mainly mediates the activation of NF-κB pathway (Au and Yeh, 2007). Indeed, study has shown that overactivation of NF-κB pathway is associated with apoptosis of alveolar epithelial type II cells (AEC2) and the development of PF (Yang et al., 2018). Besides, Ben-David et al. (2016) demonstrated that inflammatory signals regulate the expression and splicing of RIC3, thereby influencing the α7 nA-ChR mediated cholinergic anti-inflammatory pathway. Although the role of inflammation in fibrosis is controversial, it is still considered to be an important component of IPF. Recently, Kim et al. (2019) pointed out that RBM47 promotes the EMT of cells by promoting TJP1-mediated alternative splicing. Globally, EMT is considered to be one of the key mechanisms of PF. When tissues are subjected to various injuries, a series of immune signals are generated, leading to inflammation and promoting EMT. In this process, macrophages, neutrophils and other immune cells are recruited and release proinflammatory cytokines to maintain inflammation and pulmonary fibrosis (Salton et al., 2019). In conclusion, we speculate that the above three genes are closely related to the progression of pulmonary fibrosis. However, studies on RBM11 and ZNF14 in lung diseases are rare.

These results indicated that the key genes screened by bioinformatics methods were highly correlated with the occurrence and development of IPF, and had a significant correlation with the prognosis of IPF patients. Therefore, the above five key genes can provide reference for the diagnosis and treatment of IPF. We also analyzed the risk model. By drawing the time-dependent ROC curve of the prognostic model, we found that the AUC value gradually increased with the increase of time, indicating that the accuracy of our prognostic model was good. Finally, the prognostic model was verified by the GSE93606 dataset. It can be seen that the prognostic model is also applicable to this dataset, which further confirms that the prognostic risk model has a strong and stable prediction efficiency.

However, the study also has certain limitations. First, our results are based on data from existing public databases. Therefore, a large-scale, prospective, multicenter study is needed to further validate our results. Secondly, our study population is mainly from European and American populations. Therefore, our findings may not be optimal for patients from other countries and ethnicities. Finally, the correlation between some key genes and the development and progression of IPF has not been confirmed by biological experiments. In follow-up studies, experimental validation will be performed to reveal the relationship between key genes and IPF. In this way, we can determine their suitability as new diagnostic and therapeutic targets to provide a rationale for the clinical diagnosis and treatment of IPF.

## Data Availability

Publicly available datasets were analyzed in this study. This data can be found here: https://www.ncbi.nlm.nih.gov/geo/query/acc.cgi?acc&equals;GSE28042 https://www.ncbi.nlm.nih.gov/geo/query/acc.cgi
